# Piezocatalytic *in situ* production and utilization of hydrogen peroxide

**DOI:** 10.1039/d6sc05241j

**Published:** 2026-09-09

**Authors:** Zhi Li, Mingshan Zhu

**Affiliations:** a Guangdong Engineering Technology Research Center of Water Treatment Processes and Materials, College of Environment and Climate, Jinan University Guangzhou 511443 China zhumingshan@jnu.edu.cn

## Abstract

Piezocatalysis offers a route to convert mechanical energy into hydrogen peroxide (H_2_O_2_), potentially reducing the transport, storage and dosing of concentrated oxidants. However, the field remains dominated by material-centred demonstrations and reported production rates, while the functional contribution of piezogenerated H_2_O_2_ to downstream reactions remains insufficiently resolved. In this perspective, we examine piezocatalytic H_2_O_2_ production and *in situ* utilization as a coupled catalytic process. We critically discuss the mechanism and reaction pathways of piezocatalytic H_2_O_2_ production and the roles of the material and its modification strategies in regulating H_2_O_2_ production. We further evaluate the evidence supporting the involvement of *in situ* piezogenerated H_2_O_2_ in disinfection, water decontamination and medical applications and consider selective synthesis as an emerging opportunity. We believe that future progress requires force-matched controls, *operando* identification of reactive intermediates, standardized mechanical energy input, and reactor designs that couple continuous H_2_O_2_ generation with consumption. Addressing these priorities should advance piezocatalytic *in situ* H_2_O_2_ production from concept production towards practically relevant on-demand oxidation systems.

## Abstract 2

This perspective reviews piezocatalytic *in situ* production and utilization of H_2_O_2_, covering mechanisms, ORR/WOR pathways, mechanical forces, materials, modification and applications in disinfection, resistance control, water treatment and therapy.

## Graphical abstract

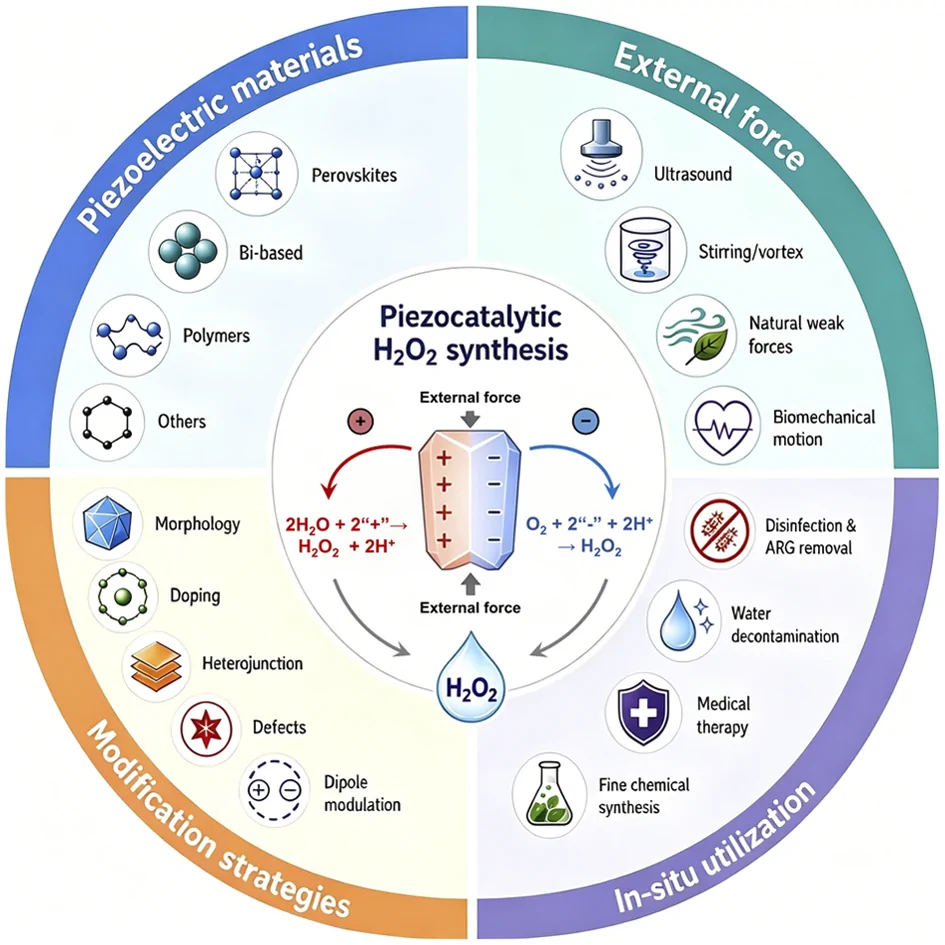

## 1. Introduction

Hydrogen peroxide (H_2_O_2_), a cornerstone chemical in modern industrial processes, plays a pivotal role in environmental remediation, energy conversion, and chemical synthesis, due to its potent oxidizing properties and eco-friendly decomposition products.^1–3^ Nowadays, the predominant industrial method for H_2_O_2_ production with the anthraquinone oxidation process suffers from significant drawbacks, including high energy consumption, reliance on complex recycling steps, and the generation of substantial organic waste.^4,5^ Alternative approaches, such as the direct synthesis of H_2_O_2_ from H_2_ and O_2_ using noble metal-based catalysts (*e.g.*, Pd, Pt, or Au), have garnered attention owing them not producing other byproducts.^6,7^ However, their practical implementation is hindered by prohibitive catalyst costs, low H_2_O_2_ selectivity and the inherent safety risks. Recently, electrocatalytic technology has been developed as a novel method for H_2_O_2_ synthesis. It utilizes renewable electricity to drive the 2-electron oxygen reduction reaction (ORR) at the cathode or 2-electron water oxidation reaction (WOR) at the anode, with tunable reaction kinetics and compatibility with sustainable energy sources.^1,8,9^ However, this method has limitations, including the competing four-electron ORR pathway and reduced overall energy efficiency induced by the oxygen evolution reaction (OER) at the anode. Recently, the activation of H_2_O_2_ generation by electron–hole mediated photocatalysis on semiconductor surfaces using solar energy with water and oxygen has emerged as an energy-efficient alternative.^2,10,11^ However, photocatalytic activity is always limited due to rapid charge recombination, poor light absorption spectra, and the use of sacrificial agents, which increases costs and complicates product separation. Therefore, there is an urgent need to develop novel green and effective methods for the efficient synthesis of H_2_O_2_.

The piezoelectric effect manifests itself in the form of non-centrosymmetric crystalline materials displaying a remarkable electrical response when subjected to mechanical forces.^12–14^ In recent years, based on this effect, researchers have begun to develop a piezocatalytic approach, an innovative way to store and convert energy from weak mechanical forces present in nature, such as wind, tide, water flow, sound, and atmospheric forces.^15,16^ This captured energy can drive atoms within the lattice of a piezoelectric material to shift, resulting in a mismatch of cation and anion centers, creating an intrinsic dipole moment.^17,18^ Moreover, the ordered summation of these dipole moments within the crystal cell produces a macroscopic built-in potential field, commonly referred to as the piezoelectric potential, accompanied by immobile and non-annihilating shielding charges.^19^ This captured energy can drive catalytic redox reactions on material surfaces, providing a compelling pathway for mechano-chemical energy conversion.

Piezocatalysis for H_2_O_2_ production represents a paradigm shift in the methodology of chemical synthesis, offering significant mechanical and operational advantages over conventional methods.^20,21^ Unlike energy-intensive anthraquinone processes, electrochemical methods that require external circuitry or unstable photocatalytic methods, piezocatalysis utilizes the ubiquitous weak mechanical energy in nature to perform the mechano-electrochemical conversion to drive the synthesis of H_2_O_2_.^2,3^ This approach takes advantage of the fact that piezoelectric materials can generate highly localized electric fields under external force. This built-in potential drives two key reactions: the 2e^−^ ORR (O_2_ + 2H^+^ + 2e^−^ → H_2_O_2_) and 2e^−^ WOR (2H_2_O → H_2_O_2_ + 2H^+^ + 2e^−^).^22,23^ Note that alternating stress-release cycles dynamically regenerate the surface charge, allowing the catalytic cycle to continue without sacrificial agents or external bias.^24^ Although challenges remain in scaling up efficiency, piezocatalytic technology fundamentally reimagines H_2_O_2_ production, including converting diffuse mechanical energy into catalytic surface chemistry with intrinsic pathway selectivity, environmental manipulation and integrative functionality, making it a cornerstone of distributed, energy chemical synthesis.

Over the past decade, piezocatalysis has attracted growing interest in energy conversion and environmental remediation. Recent reviews have summarized its fundamental mechanisms, material categories, modification strategies and major applications,^25–28^ while some reviews have further discussed piezopolarization-assisted H_2_O_2_ production.^29,30^ However, existing studies have focused predominantly on catalyst development and H_2_O_2_ generation, with considerably less attention given to its *in situ* utilization. Several critical questions therefore remain unresolved, including whether piezogenerated H_2_O_2_ serves as the functional intermediate in downstream reactions and how it is subsequently activated or selectively consumed. Here, we discuss the mechanistic basis, material design and mechanical inputs governing piezocatalytic H_2_O_2_ production, together with its prospective *in situ* utilization in environmental remediation, biomedicine and selective chemical synthesis. Particular attention is given to the evidence linking H_2_O_2_ generation to downstream functions, the comparability of reported performance metrics, and the practical constraints associated with energy input, reaction selectivity, catalyst stability and reactor scale-up. This perspective aims to advance the field beyond material-centred proof-of-concept studies towards mechanistically verifiable and practically relevant H_2_O_2_ production and utilization systems.

## 2. Basic principles

### 2.1 Mechanism of piezocatalysis

Two complementary models are commonly used to describe piezocatalysis. In the semiconductor band model, mechanical strain generates an alternating piezopotential that induces band bending and drives electrons and holes toward opposite polar surfaces, thereby promoting charge separation and interfacial redox reactions (Fig. 1a).^24^ Periodic strain is required to prevent internal mobile charges from screening the piezopotential and to continuously regenerate surface-reactive charges. These charges are dissipated through capacitive and faradaic processes. However, the origin and excitation of charge carriers under mechanical stimulation remain under debate.

**Fig. 1:**
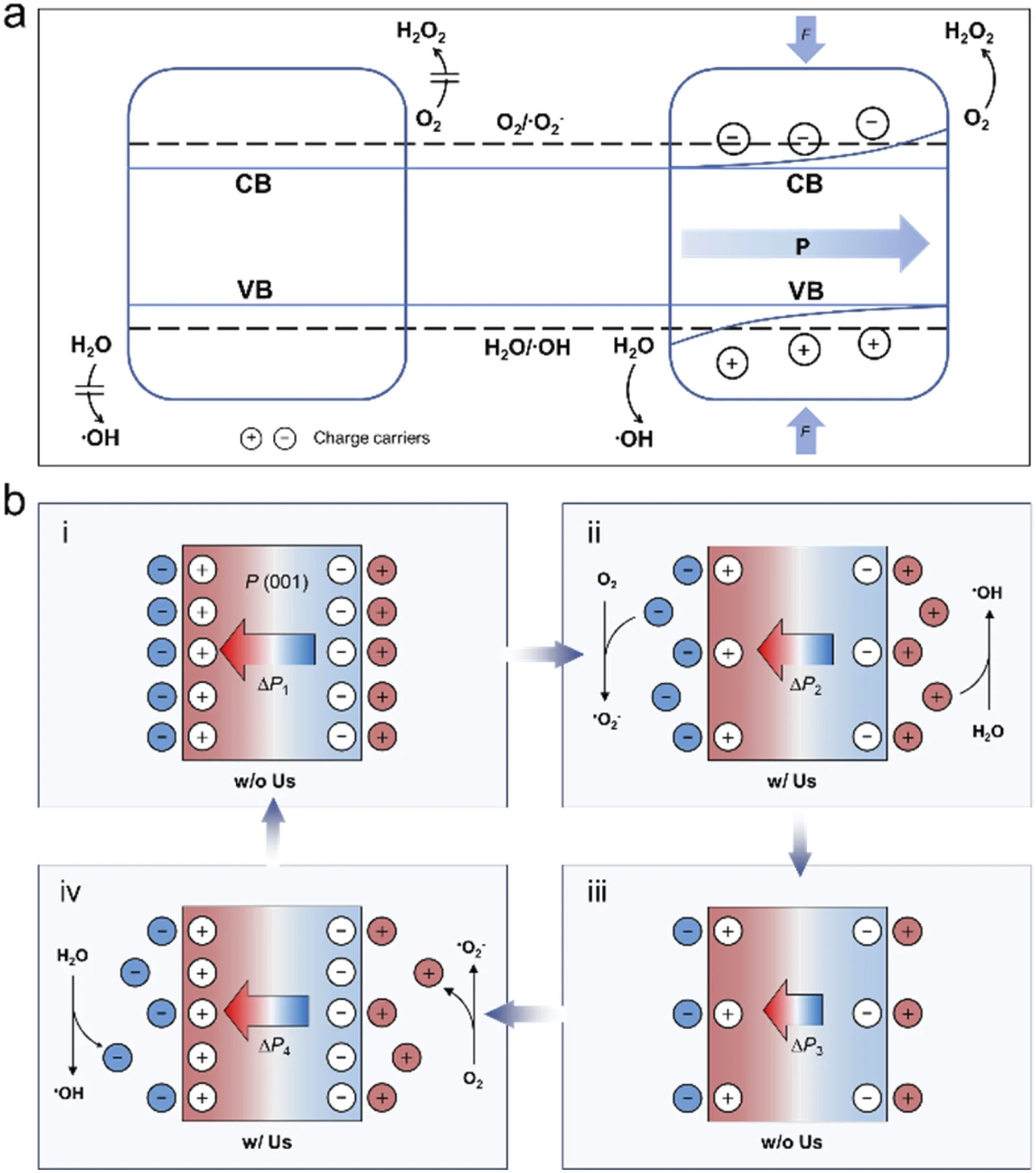
Piezocatalysis mechanisms for H_2_O_2_ production by energy band theory mechano-induced charge carrier generation (a). Piezocatalysis based on the screening charge effect (b). (i) The pristine electrostatic balance state. (ii) The release of surface screening charges under compressed strain to trigger surface redox reactions. (iii) A transient electrostatic balance state under maximum strain. (iv) The adsorption of screening charges from the electrolyte upon revival of the polarization and surface reactions.

The screening-charge model instead emphasizes polarization-dependent charge exchange at the solid–liquid interface.^31,32^ At equilibrium, polarization-bound charges are compensated for by oppositely charged species adsorbed on the polar surfaces (Fig. 1b(i)). Compression changes the polarization and disrupts this equilibrium, releasing screening charges that can participate in surface redox reactions (Fig. 1b(ii)). A new equilibrium is then established under maximum strain (Fig. 1b(iii)). When the stress is released, polarization recovers and compensating charges are re-adsorbed from the electrolyte, enabling complementary reactions (Fig. 1b(iv)). Tensile stress induces the reverse cycle. Thus, periodic ultrasonication, oscillation, or stirring can sustain polarization-mediated charge release and adsorption, provided that the resulting surface potential is sufficient to drive the target reaction.

### 2.2 Mechanism of piezocatalytic H_2_O_2_ synthesis

Piezocatalytic H_2_O_2_ production mainly proceeds through the oxygen reduction reaction (ORR) and water oxidation reaction (WOR) (Fig. 2a).^2,33^ In the ORR pathway, piezoelectrically generated electrons or positive screening charges reduce adsorbed O_2_ through either a sequential one-electron pathway involving ˙O_2_^−^ and HO_2_˙^−^ intermediates or a direct two-electron pathway:

1 O_2_ + e^−^ → ˙O_2_^−^

2 ˙O_2_^−^ + H^+^ → HO_2_˙^−^

3 HO_2_˙^−^ + e^−^ → HO_2_^−^

4 HO_2_^−^ + H^+^ → H_2_O_2_

5 O_2_ + e^−^ → ˙O_2_^−^ → ˙OOH → H_2_O_2_

6 O_2_ + 2H^+^ + 2e^−^ → H_2_O_2_

**Fig. 2:**
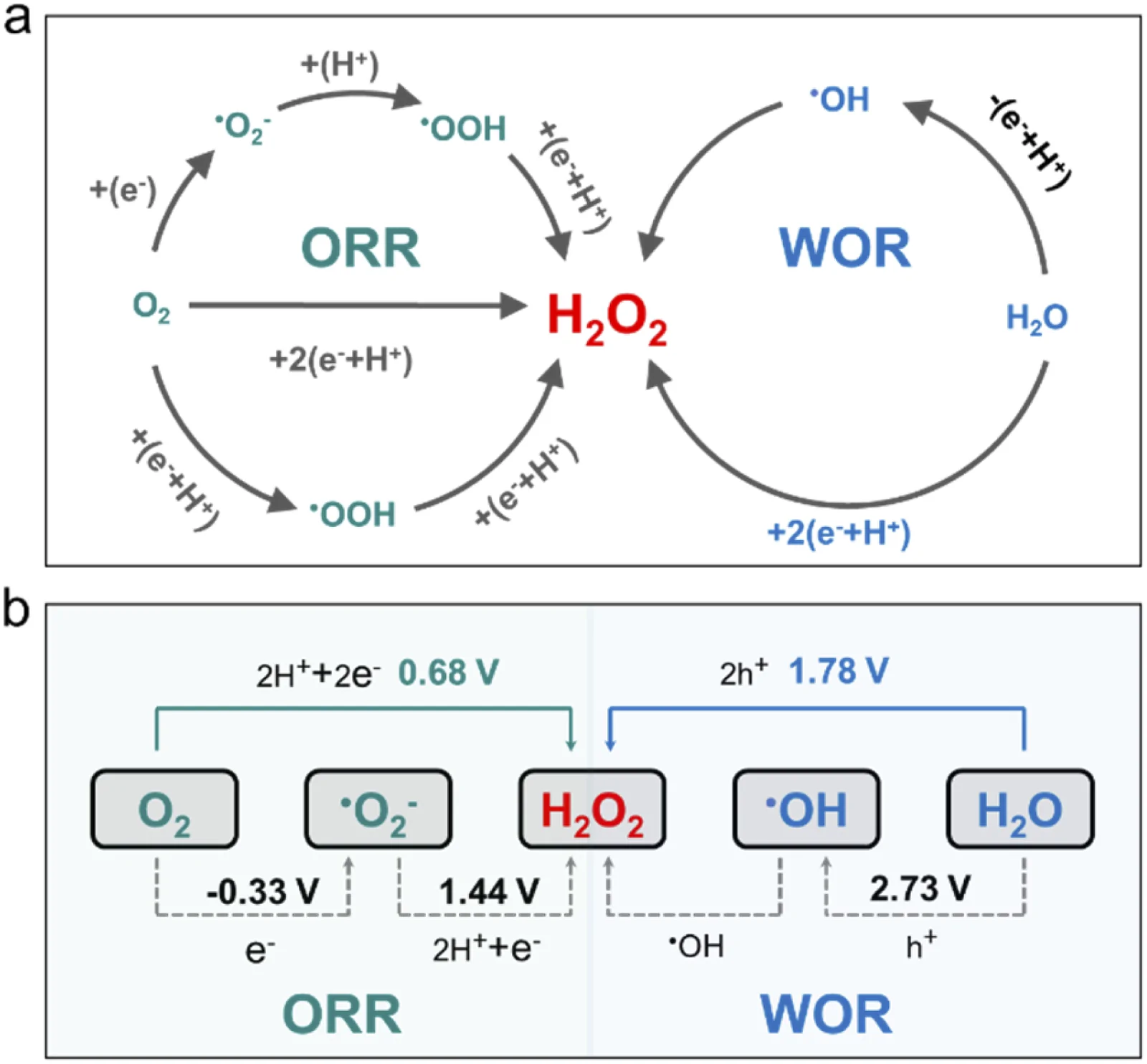
H_2_O_2_ production *via* the water oxidation reaction (WOR) and oxygen reduction reaction (ORR) (a). H_2_O_2_ formation pathway *vs.* NHE scale (b).

Eqn (1)–(5) illustrate the process of H_2_O_2_ formation *via* an indirect two-step single-electron reduction pathway, while eqn (6) illustrates the pathway for H_2_O_2_ formation *via* direct one-step two-electron reduction. In this process, O_2_ reacts directly with two H^+^ ions to form H_2_O_2_*via* two-electron reduction.

In the WOR pathway, piezoelectrically generated holes or positive screening charges oxidize H_2_O to ˙OH (eqn (7) and (8)), followed by radical coupling to form H_2_O_2_ and an overall two-electron WOR to produce H_2_O_2_ in eqn (9) (Fig. 2b).^34,35^

7 H_2_O + h^+^ → ˙OH + H^+^

8 2˙OH → H_2_O_2_

9 2H_2_O + 2h^+^ → H_2_O_2_ + 2H^+^

Coupling the WOR with the ORR gives the overall reaction in eqn (10):

10 2H_2_O + O_2_ → 2H_2_O_2_

The piezocatalytic reaction of H_2_O and O_2_ to produce hydrogen peroxide is an uphill reaction, with a standard Gibbs free energy change Δ*G*_0_ of 117 kJ mol^−1^.

## 3. Overview of piezocatalytic H_2_O_2_ production

### 3.1 External force used

Currently, a variety of external forces are used to activate piezoelectric catalysis, including ultrasonic force, stirring and natural weak mechanical force (Fig. 3). A detailed classification is presented below.

**Fig. 3:**
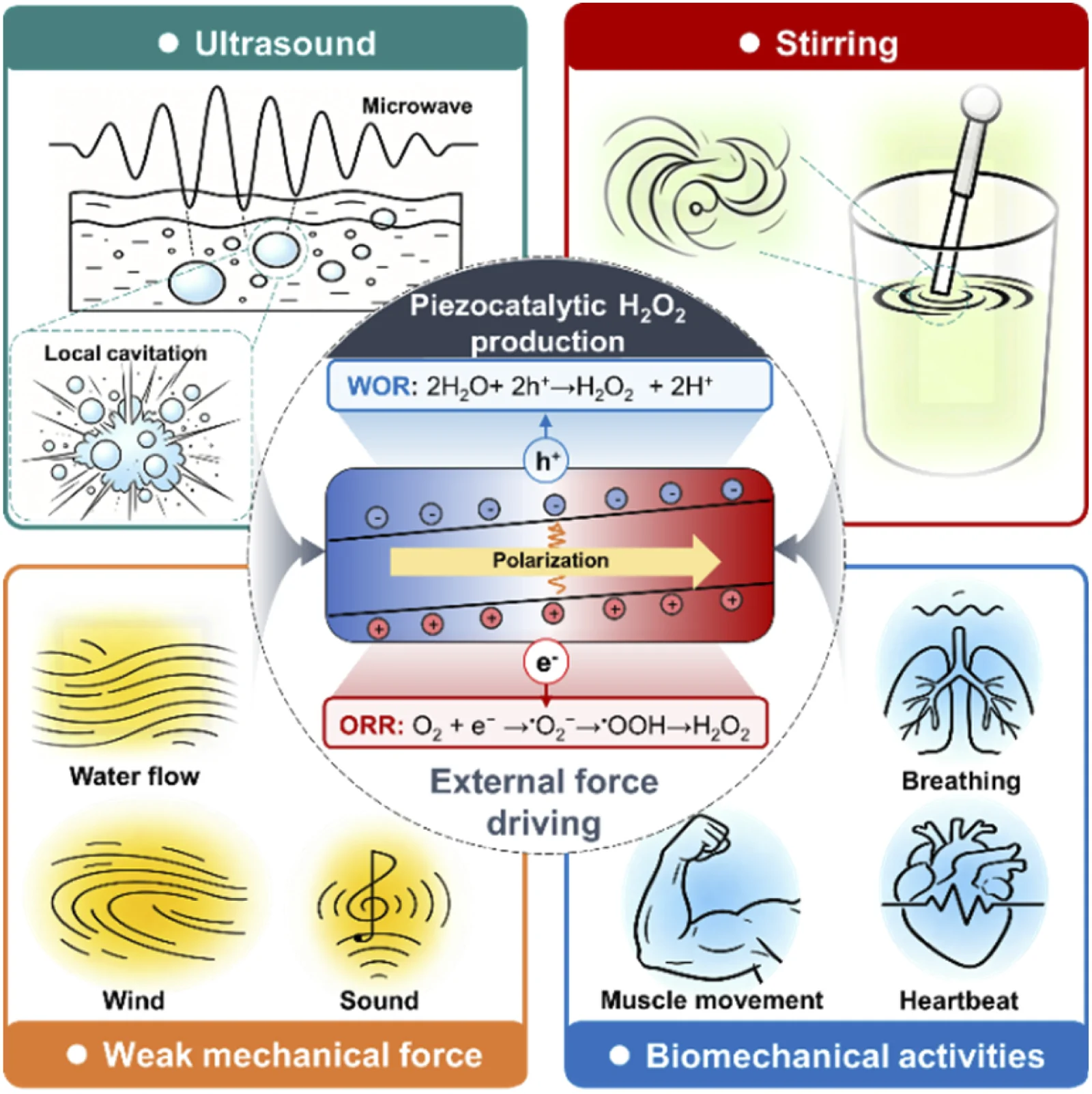
Piezocatalytic H_2_O_2_ production with external force, including ultrasound, stirring, weak mechanical forces (water flow, wind and sound) and biomechanical activities (breathing, heartbeat, and muscle movement).

#### 3.1.1 Ultrasonic force

Ultrasound is the most widely used mechanical input. When high-frequency sound waves (>20 kHz) propagate through a liquid, the formation and collapse of cavitation bubbles generate shock waves and microjets that repeatedly deform dispersed piezoelectric particles.^36–43^ The resulting alternating piezopotential promotes charge separation and drives the ORR and/or WOR toward H_2_O_2_ formation. Under ultrasound alone, carbon quantum dot-anchored C_3_N_5_ (CQDs-C_3_N_5_) produced H_2_O_2_ at 5025 µmol g^−1^ h^−1^ at 40 kHz and 100 W,^22^ while CTN-1 achieved 4115 µmol g^−1^ h^−1^ at 40 kHz and 150 W.^23^ These systems did not require simultaneous light irradiation or an externally added oxidant. Nevertheless, the nominal power of an ultrasonic bath should not be treated as the acoustic power delivered to the reaction suspension.

#### 3.1.2 Stirring

Stirring produces fluid shear and vortices that can deform piezoelectric particles, particularly flexible nanosheets, nanowires and supported nanoarrays.^44,45^ Although stirring generally provides weaker mechanical stress than ultrasound, it is operationally simple and potentially scalable. In a related contact-electro-catalytic system, continuous agitation with a poly(tetrafluoroethylene) stir bar combined with ultrasonication achieved an H_2_O_2_ production rate of 256.6 µM h^−1^ under ambient conditions without dispersed catalyst particles or sacrificial agents. Because ultrasound was applied simultaneously and no piezoelectric catalyst was used, this system should be classified as stirring-ultrasound-assisted contact-electro-catalysis rather than purely stirring-driven piezocatalysis.

#### 3.1.3 Natural weak mechanical forces

Natural weak mechanical forces refer to the low-intensity, low-frequency mechanical energies ubiquitously present in the environment, including water flow (rivers, tides, waves, and microdroplets), wind, sound, and biomechanical activities such as breathing, heartbeat, and muscle movement.^46,47^ These energy sources are renewable, cost-free, and widely distributed, but their low energy density has traditionally made them difficult to harness for chemical reactions. For example, water microdroplets provide another mechanically enabled but non-piezocatalytic pathway.^48^ Atomizing pure water into airborne droplets approximately 1–20 µm in diameter produced about 30 µM H_2_O_2_ without added reagents, catalysts, applied voltage or irradiation. H_2_O_2_ production increased with decreasing droplet size, and recombination of interfacially generated ˙OH was proposed as its origin.

### 3.2 Piezoelectric materials for H_2_O_2_ production

This section primarily summarizes the mainstream materials, including perovskites, bismuth-based materials, organic polymers and others, which were used for the piezocatalytic synthesis of H_2_O_2_ (Fig. 4).

**Fig. 4:**
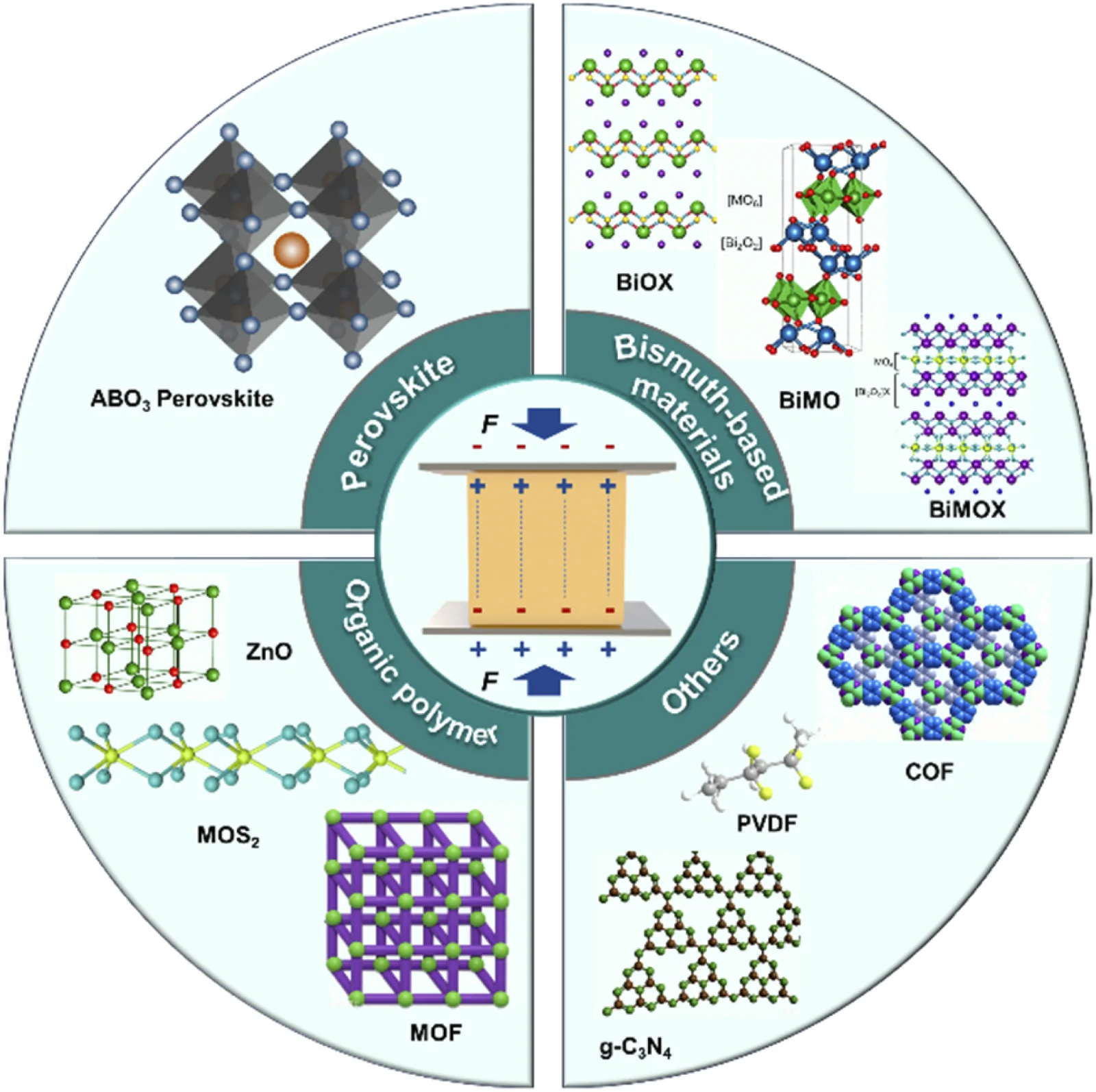
Piezocatalytic H_2_O_2_ production with materials, including perovskites, bismuth-based materials, organic polymers and others.

#### 3.2.1 Perovskites

Perovskite materials, characterized by the general formula ABX_3_ (where A and B are cations of different sizes and X is an anion, typically oxygen or halogen), represent one of the most important and extensively studied classes of piezoelectric materials for H_2_O_2_ production.^49,50^ In inorganic oxide perovskites such as BaTiO_3_, the crystal structure consists of a network of corner-sharing BO_6_ octahedra, with the A-site cation occupying the twelve-fold coordination cavities.^51^ The origin of piezoelectricity in perovskites lies in their non-centrosymmetric crystal structure, typically tetragonal, rhombohedral, or orthorhombic phases, where the B-site cation is displaced from the center of the BO_6_ octahedron, creating a permanent dipole moment. Under external mechanical stress, this displacement changes, altering the charge distribution and generating a piezoelectric potential across the material.^52^ This piezopotential can drive the two-electron ORR or WOR to produce H_2_O_2_. The ability to tune the A- and B-site elements enables the development of a vast family of perovskites with tailored piezoelectric and semiconductor properties, making them highly effective for piezocatalytic H_2_O_2_ synthesis.

#### 3.2.2 Bismuth-based materials

Bismuth-based piezoelectric materials constitute a diverse family of compounds that have attracted considerable attention for H_2_O_2_ production due to their low toxicity, earth abundance, and unique crystal structures. These materials include layered bismuth-based (LBB) compounds such as BiOIO_3_, Bi_4_Ti_3_O_12_, Bi_2_O_2_(OH)(NO_3_), BiOCl, BiOBr, and BiOI, as well as perovskite-type bismuth ferrite (BiFeO_3_).^53–56^ The key structural feature common to many bismuth-based piezoelectrics is the presence of [Bi_2_O_2_]^2+^ layers interleaved with halogen atoms or other anionic groups. The stereochemical activity of the Bi^3+^ 6s^2^ lone pair electrons drives asymmetric displacement, resulting in strong spontaneous polarization and excellent piezoelectric properties even without external poling. Under mechanical stress, the non-centrosymmetric crystal lattice undergoes deformation, separating positive and negative charge centers to generate a piezopotential, which can selectively reduce O_2_ to H_2_O_2_*via* a two-electron pathway.

#### 3.2.3 Organic polymers

Organic polymer piezoelectric materials mainly include graphitic carbon nitride (g-C_3_N_4_), covalent organic frameworks (COFs), polyvinylidene fluoride (PVDF) and its copolymers, and other polar polymers such as poly(tetrafluoroethylene) (PTFE).^19,23,57–64^ The piezoelectric effect in these polymers originates from the polar nature of their molecular chains or asymmetric structural units. For instance, PVDF exhibits piezoelectricity due to its β-phase (all-*trans* conformation) where all dipole moments of CF_2_ groups align in the same direction, creating a non-centrosymmetric structure. Under mechanical stress (bending, stretching, or vibration), these dipoles reorient, generating a piezopotential. Similarly, g-C_3_N_4_ possesses non-centrosymmetric triangular nanopores and a flexoelectric effect, giving it a significant piezoelectric response with a calculated e_11_ coefficient of 0.758 C m^−2^.^65^ COFs and other nitrogen-rich organic networks can also be designed with asymmetric topologies or polar functional groups to achieve piezoelectricity. These organic polymer materials offer unique advantages for H_2_O_2_ production, including light weight, high flexibility, easy processability, good biocompatibility, and molecular designability. Under external mechanical forces (*e.g.*, ultrasound, stirring, and water flow), their lattice or molecular chains undergo deformation, separating positive and negative charge centers to form a piezopotential, which drives free charges to migrate to the material surface and trigger the two-electron ORR or WOR for H_2_O_2_ synthesis.

#### 3.2.4 Others

Beyond perovskites, bismuth-based compounds, and organic polymers, several other material families have been developed as effective piezocatalysts for H_2_O_2_ production. These include wurtzite-structured zinc oxide (ZnO), two-dimensional transition metal dichalcogenides (TMDs) such as MoS_2_, WS_2_, and WSe_2_, and metal–organic frameworks (MOFs).^66–68^ ZnO crystallizes in the hexagonal wurtzite structure with spontaneous polarization along the *c*-axis; when subjected to mechanical stress, the displacement of Zn^2+^ and O^2−^ ions generates a piezopotential that can reduce O_2_ to H_2_O_2_.^69^ Two-dimensional TMDs with odd numbers of layers (*e.g.*, monolayer 2H-MoS_2_) exhibit strong in-plane piezoelectricity due to broken inversion symmetry, and their high flexibility allows efficient energy harvesting from low-frequency mechanical disturbances for H_2_O_2_ synthesis.^70^ MOFs with non-centrosymmetric structures, such as ZIF-8 and UiO-66-NH_2_, have recently emerged as customizable piezocatalysts due to their ultrahigh surface area and tunable porosity.^71^ Furthermore, symmetry-breaking strategies can induce piezoelectricity in intrinsically centrosymmetric MOFs, expanding the material palette for piezocatalytic H_2_O_2_ production.

### 3.3 Material modification strategies

This section primarily summarizes the mainstream modification strategies of materials, including morphology tuning, elemental doping, heterojunction regulation, defect control and dipole modulating, which are used for piezocatalytic H_2_O_2_ production (Fig. 5).

**Fig. 5:**
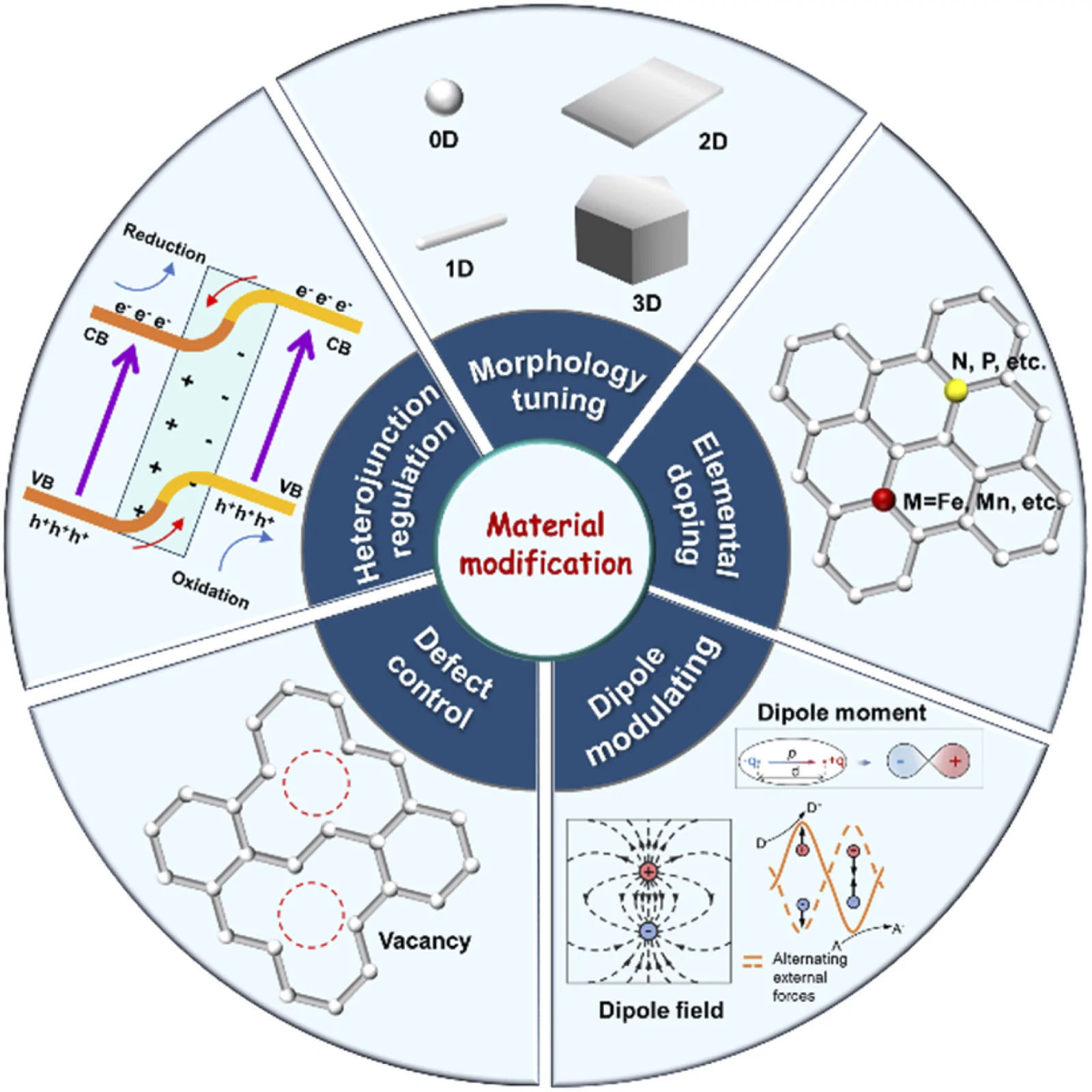
Modification strategies of piezocatalytic materials for H_2_O_2_ production, including morphology tuning, elemental doping, heterojunction regulation, defect control and dipole modulating.

#### 3.3.1 Morphology tuning

Morphology tuning controls the size, shape, and architecture of piezoelectric materials to improve their response to mechanical stress. High-aspect-ratio structures, including nanofibers, nanorods, nanosheets, and hollow nanotubes, undergo greater bending or torsion than bulk particles, thereby producing a stronger piezopotential. Low-dimensional structures also expose more active sites and shorten the migration distance of piezo-induced charges. Their activity therefore depends not only on surface area, but also on how efficiently a given structure concentrates and transfers mechanical stress. These effects jointly improve mechanical-energy conversion, carrier utilization, and interfacial H_2_O_2_ synthesis. For example, NiFe_2_O_4_ nanofibers containing surface oxygen vacancies increased piezocatalytic H_2_O_2_ generation by 39% relative to bulk NiFe_2_O_4_ in pure water, because the fibrous structure and vacancies promoted charge transfer and O_2_ activation.^72^ Core–shell BiOCl@BiPO_4_ nanoplates combined a deformable plate-like morphology with a heterojunction and abundant Lewis and Brønsted acid sites, delivering 884.7 µmol g^−1^ h^−1^, compared with 392.6 and 558.3 µmol g^−1^ h^−1^ for BiOCl and BiPO_4_, respectively.^73^ Hollow-tubular g-C_3_N_4_ also produced 262 µmol g^−1^ h^−1^ without a cocatalyst, exceeding that of nanosheets and hollow nanospheres by 1.5 and 6.2 times.^74^

#### 3.3.2 Elemental doping

Elemental doping introduces foreign atoms into substitutional or interstitial lattice sites. The difference in the ionic radius between the dopant and host atom produces lattice distortion and may generate vacancies, increasing structural asymmetry and the piezoelectric response. Doping can also regulate band positions, carrier density, intermediate states, and electron spin, thereby improving charge separation and changing the H_2_O_2_ formation pathway. An appropriate dopant can consequently shift oxygen reduction from a stepwise single-electron route toward a kinetically more favorable direct 2e^−^ route. Among Co-BiOBr samples, 10% Co-BiOBr exhibited the highest H_2_O_2_ production rate of 251.3 µmol g^−1^ h^−1^. Co incorporation modified its morphology and electronic states, enhanced charge transfer, and facilitated dual-channel H_2_O_2_ generation.^75^ Cd_0.5_Zn_0.5_S nanobranches produced 21.9 µmol g^−1^ h^−1^ H_2_O_2_ under ultrasound without sacrificial agents. Their branched morphology favored mechanical-energy conversion, while H_2_O_2_ formed through the sequential two-step single-electron ORR.^76^ Fe doping in Bi_3_TiNbO_9_ further coupled piezoelectricity with spin polarization. Fe-induced off-centering of [TiO_6_]/[NbO_6_] octahedra strengthened the piezoresponse, while magnetic-field-enhanced spin polarization increased carrier mobility and lowered the barrier for the direct one-step 2e^−^ ORR. Consequently, Fe-doped Bi_3_TiNbO_9_ reached 1421.2 µmol g^−1^ h^−1^ H_2_O_2_, 10.4 times that of pristine Bi_3_TiNbO_9_.^77^

#### 3.3.3 Heterojunction regulation

Heterojunction regulation couples two or more semiconducting or piezoelectric components. Differences in their work functions and band positions induce charge redistribution and an interfacial electric field. Under mechanical stress, the piezopotential promotes bulk charge separation, while the interfacial field directs electrons and holes toward different reaction sites. This dual-field effect suppresses recombination and preserves the redox potentials required for H_2_O_2_ synthesis. Z-scheme interfaces may additionally mitigate polarization screening by preventing mobile carriers from prematurely compensating for polarization charges. They can also spatially separate WOR and ORR sites, allowing water oxidation and oxygen reduction to contribute simultaneously without sacrificing the redox capacity of either component. A ZnO/Zr-MOL Z-scheme heterojunction achieved 13.21 mmol g^−1^ h^−1^ in pure water and ambient air by regulating band bending and retaining long-lived charge separation.^78^ A 2D/2D BiOIO_3_/carbon nitride heterojunction combined an interfacial electric field with bulk piezo-polarization, enabling the 2e^−^ WOR on BiOIO_3_ and 2e^−^ ORR on carbon nitride. It produced 259.8 µM H_2_O_2_ within 30 min in pure water and air without sacrificial agents or aeration.^79^ Similarly, BaTiO_3_@COF provided separate microsites for water and oxygen activation and generated 240.7 µmol g^−1^ h^−1^ through dual-pathway synthesis.^80^

#### 3.3.4 Defect control

Defect control regulates oxygen, metal, sulfur, or other lattice vacancies. These defects induce local distortion, enhance structural asymmetry, and may increase the piezopotential. They also act as adsorption or charge-trapping sites, facilitating O_2_ and H_2_O activation while suppressing carrier recombination. Defect engineering can even induce piezoelectricity in centrosymmetric materials by breaking local inversion symmetry; however, excessive defects may reduce crystallinity and piezoelectricity. Defect concentration must therefore be optimized to balance polarization enhancement, reactant adsorption, charge trapping, and structural stability. Under ultrasound, cobalt-loaded ZnO generated 1.3 mM H_2_O_2_ and showed increasing production over repeated cycles, reaching 1.8 mM because piezoelectric electrons dynamically generated defects that promoted O_2_ adsorption and activation. The catalyst also retained activity after prolonged inactivity, and performance loss caused by metal dissolution could be reversed by adjusting pH.^20^ Oxygen-defective g-C_3_N_4_ (CN-40) produced 671 µmol g^−1^ h^−1^ in pure water without sacrificial agents, four times the yield of pristine g-C_3_N_4_. Oxygen incorporation broke the symmetry of tri-*s*-triazine units, strengthened polarization, increased carrier density, and promoted coupled water oxidation and oxygen reduction.^81^ Oxygen vacancies also induced piezoelectricity in centrosymmetric SrFeO_3−*x*_, producing a surface potential of up to 93 mV and an H_2_O_2_ concentration of 1821 µM under ultrasonic vibration.^82^

#### 3.3.5 Dipole modulation

Dipole modulation increases the net polarization and piezopotential generated under mechanical stress. Phase-boundary engineering lowers the energy barrier for polarization rotation, whereas crystal orientation and ferroelectric poling align dipoles along the preferred direction. Asymmetric molecular units, functional groups, and vacancies can also create permanent local dipoles. Operation near a suitable phase transition may further increase dielectric and piezoelectric responses, although temperature-dependent effects require careful control. These approaches strengthen the driving force for charge separation and surface redox reactions. In OCN-460, carbon vacancies and oxygen doping created asymmetric triazine units and a spontaneous polarization field, promoting directional charge transfer. Combined with its ultrathin porous structure, OCN-460 achieved a piezo-photocatalytic H_2_O_2_ production rate of 19.30 mmol g^−1^ h^−1^ and retained 2.87 mmol g^−1^ h^−1^ in pure water.^83^ Similarly, the asymmetric triazole units of C_3_N_5_ generated permanent dipoles and promoted Pauling-type O_2_ adsorption through an indirect 2e^−^ pathway. Under simulated sunlight and ultrasound, C_3_N_5_ produced H_2_O_2_ at 3809.5 µmol g^−1^ h^−1^.^57^

Based on all the above materials and their modification strategies, the current yields of H_2_O_2_ synthesized *via* piezoelectric catalysis are summarized in Table 1, including the catalyst, mechanical input, catalyst dosage, liquid volume, medium, sacrificial reagent, normalized H_2_O_2_ production rate, stability, and references.

**Table 1:** Piezocatalytic H_2_O_2_ production rates of the representative recently reported work

Catalyst	Mechanical input	Catalyst/liquid volume	Medium/additive	Sacrificial reagent	Normalized H_2_O_2_ rate (µmol g^−1^ h^−1^)	Stability	Ref.
BCZT-0.5	Ultrasound (40 kHz, 180 W)	50 mg/50 mL	Water/O_2_	10 vol% Ethanol	692	4 cycles	84
MBTO-I (MXene-coupled, I-doped Bi_4_Ti_3_O_12_)	Ultrasound (40 kHz, 100 W)	5 mg/30 mL	Pure water/air, 15 °C water bath	None	5890	6 cycles	85
COF/BaTiO_3_	Ultrasound (300 W)	50 mg/100 mL	Pure water/air	None	240.7	5 cycles	80
Ultrathin Bi_4_Ti_3_O_12_ nanosheets with surface O-vacancies	Ultrasound (40 kHz, 300 W)	50 mg/100 mL	Water/air	10 vol% Ethanol	1611.2	4 cycles	86
C-BiOCl nanosheets	Ultrasound with stirring (40 kHz, 100 W)	20 mg/100 mL	Water/air	None	2110	Continuous operation for 24 h	87
Core–shell BiOCl@BiPO_4_ nanoplates	Ultrasound (40 kHz, 100 W)	10 mg/20 mL	Water/O_2_	None	884.7	Continuous operation for 12 h	73
CQDs-C_3_N_5_	Ultrasound (40 kHz, 100 W)	2 mg/10 mL	Water/air	None	5025	5 cycles	22
CTN-1 (covalent triazine-based nanotube)	Ultrasound (40 kHz, 100 W)	2 mg/10 mL	Water/air	None	4115	5 cycles	23
TiO_2_/ZnO	Ultrasound (40 kHz, 360 W)	5 mg/50 mL	Water/air	None	2078.14	4 cycles	67
UiO-66(Hf_3_Zr_3_)	Ultrasound (320 W, 40 kHz)	20 mg/100 mL	Water/air, 25 °C circulating water	None	4800	Continuous operation for 15 h	71
NiFe_2_O_4_ nanofibers with surface O-vacancies	Ultrasound (150 W, 40 kHz)	50 mg/80 mL	Water/air	None	456	6 cycles	72
Hollow-nanotube g-C_3_N_4_	Ultrasound with stirring (150 W, 45 kHz)	100 mg/100 mL	Water/air	None	262	4 cycles	74
10% Co-BiOBr	Ultrasound (40 kHz, 200 W)	25 mg/100 mL	Water/air	5 vol% Ethanol	251.3	3 cycles	75
Cd_0.5_Zn_0.5_S nanobranches	Ultrasound (40 kHz, 120 W)	50 mg/100 mL	Water/air	None	21.9	4 cycles	76
Fe-doped Bi_3_TiNbO_9_	Ultrasound with stirring (300 W, 40 kHz)	50 mg/50 mL	Water/air	20 vol% Ethanol	1421.2	5 cycles	77
ZnO/Zr-MOL	Ultrasound (200 W, 40 kHz)	25 mg/50 mL	Water/air	None	660.5	10 cycles	78
BiOIO_3_/carbon nitride	Ultrasound (120 W, 40 kHz)	25 mg/50 mL	Water/air	None	1039.2	4 cycles	79
Co-loaded ZnO (CZO)	Ultrasound	[Catalyst] = 0.6 g L^−1^	Water/air	None	2167	4 cycles	20
CN-40 (O-doped/defective g-C_3_N_4_)	Ultrasound (400 W, 45 kHz)	50 mg/50 mL	Water/air	None	671	4 cycles	81
SrFeO_3−*x*_	Ultrasound (150 W, 40 kHz)	40 mg/80 mL	Water/air	None	1821	5 cycles	82
SnO-OVs	Ultrasound (120 W, 40 kHz)	25 mg/50 mL	Water/air	None	512.29	4 cycles	88

## 4. *In situ* utilization

### 4.1 Disinfection and antibiotic resistant gene degradation

The widespread occurrence of pathogenic microorganisms and antibiotic-resistant bacteria (ARB) together with antibiotic resistance genes (ARGs) in water bodies poses a serious threat to public health and ecological safety, while conventional disinfection methods such as chlorination and UV irradiation often produce toxic by-products, suffer from limited penetration, and show poor efficacy against ARB and ARGs.^89^ Piezocatalysis offers a compelling alternative because it can generate reactive oxygen species (ROS), including H_2_O_2_, hydroxyl radicals (˙OH), superoxide anions (˙O_2_^−^) and singlet oxygen (^1^O_2_), *in situ* under mechanical agitation (ultrasound, water flow or stirring), without the need for external chemical oxidants. These ROS efficiently disrupt bacterial cell membranes, damage DNA and proteins, and also degrade free extracellular ARGs, thereby providing simultaneous disinfection and resistance-gene elimination. For example, a PTFE-ZnO nanofiber membrane under ultrasonic activation achieved complete ARB inactivation in the retentate zone and reduced total ARG abundance from ∼1 × 10^3^ to ∼5 × 10^6^ copies mL^−1^ in the permeate, with horizontal gene transfer frequency lowered by seven orders of magnitude (Fig. 6a).^90^ In another study, PNN-PT perovskite piezocatalysts possessing a high piezoelectric coefficient (*d*_33_ ≈ 527 pC N^−1^) and a positive surface potential (+26.8 mV) inactivated >99.9% of methicillin-resistant *Staphylococcus aureus* and *Pseudomonas aeruginosa* within 15 min under ultrasound, and the activity was retained over 16 cycles in real wastewater (Fig. 6b).^91^ Additionally, CQD-C_3_N_5_ piezocatalysts achieved an outstanding H_2_O_2_ synthesis rate of 5025 µmol g^−1^ h^−1^ through an H-bond-driven proton-coupled electron transfer process, and the constructed piezo-self-Fenton system efficiently removed chemical oxygen demand, antibiotic-resistant bacteria and antibiotic resistance genes from oxytetracycline-rich wastewater (Fig. 6c).^22^ Even gentle water flow has been exploited: MoS_2_ nanosheets piezocatalytically produced H_2_O_2_ and ROS under natural water flow, inactivating both Gram-negative and Gram-positive bacteria attached on microplastics, demonstrating a self-powered, energy-free disinfection approach (Fig. 6d).^92^

**Fig. 6:**
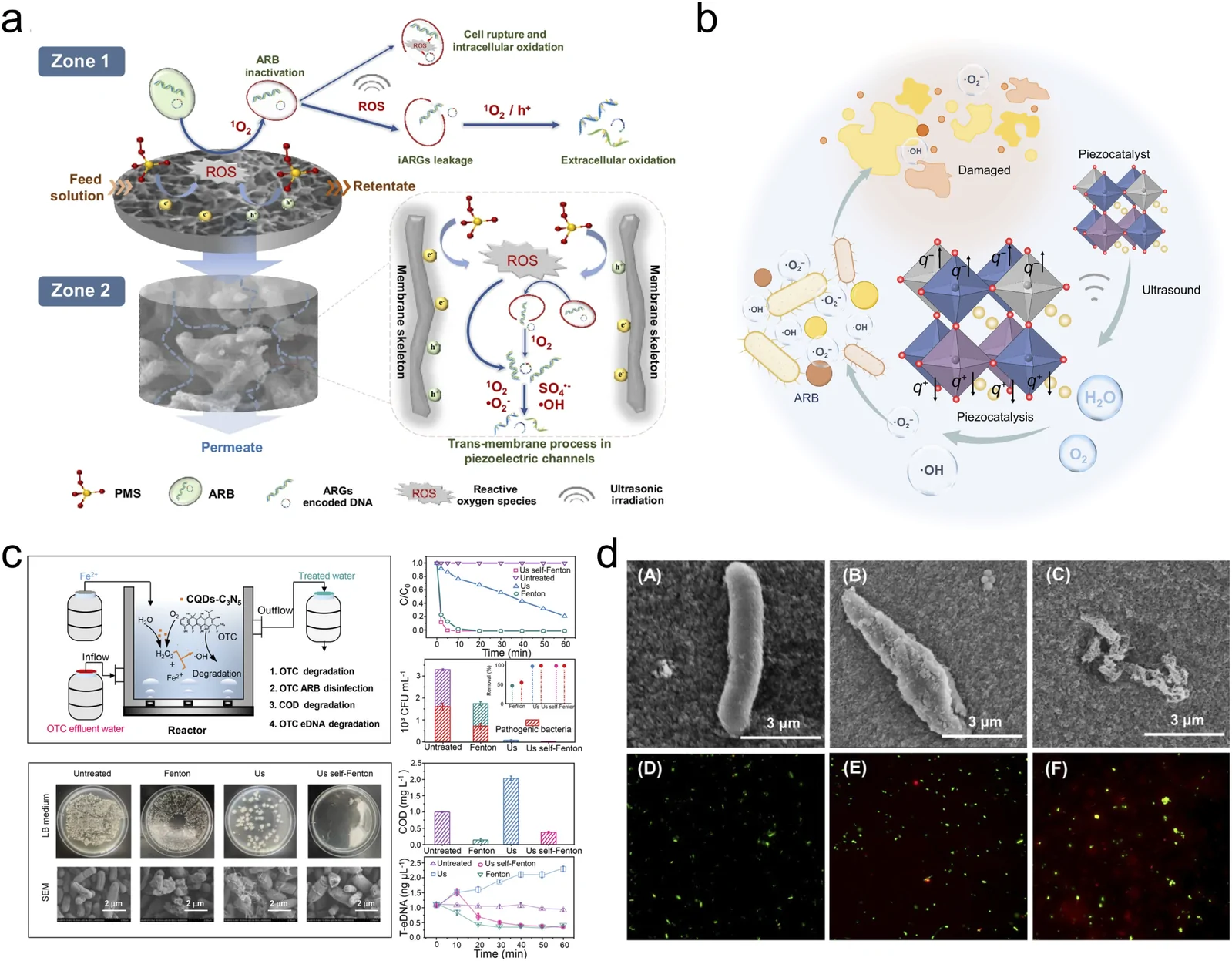
Schematic illustration of the configuration of a cross-flow piezoelectric membrane filtration device (a). Reproduced with permission from ref. 90 Copyright 2024 Springer Nature. Schematic diagram of an ultrasound-activated perovskite piezocatalyst for efficient inactivation of antibiotic-resistant bacteria in a complex wastewater system (b). Reproduced with permission from ref. 91 Copyright 2026 Springer Nature. Schematic diagram of the continuous-flow reactor and OTC effluent water quality assessment process, degradation efficiencies of OTC, LB medium and SEM images of plate counting of ARB with different treatments, removal efficiencies of total ARB and intestinal pathogenic bacteria, and COD and total eDNA in real OTC water (c). Reproduced with permission from ref. 22 Copyright 2025 Wiley. SEM images of *E. coli* from untreated and treated samples and fluorescence microscopic images of *E. coli* in the blank system, 50 µm PVC-mediated system, and 50 µm PVC/MoS_2_-mediated system for 20 min (d). Reproduced with permission from ref. 92 Copyright 2022 American Chemical Society.

### 4.2 Water decontamination

Industrial effluents, agricultural runoff and domestic sewage contain a wide variety of recalcitrant organic pollutants such as dyes, antibiotics, pesticides and phenols, which are difficult to mineralize by conventional biological or physical methods, while traditional advanced oxidation processes (AOPs) rely on externally added H_2_O_2_ or other strong oxidants, leading to high costs and secondary pollution. Piezocatalysis enables the direct conversion of mechanical energy into chemical energy, producing H_2_O_2_ and multiple ROS *in situ*, which can attack organic pollutants directly or *via* Fenton-like reactions and persulfate activation, achieving efficient degradation and even complete mineralization over a broad pH range without external oxidant addition.^42^ For instance, cobalt-loaded ZnO (CZO) piezocatalysts generated dynamic oxygen vacancies under ultrasound, which increased the H_2_O_2_ yield from 1.3 mM to 1.8 mM after four cycles, while achieving 98.7% phenol removal and 50.9% TOC removal, and the system was also effective for other pollutants such as rhodamine B, methylene blue, 4-chlorophenol and sulfadiazine.^20^ A BiOIO_3_/γ-FeOOH (BF1.5) piezocatalyst produced H_2_O_2_ through both two-electron water oxidation and two-electron oxygen reduction; the electron-rich FeOOH surface created an acidic microenvironment that activated the *in situ*-generated H_2_O_2_ to ˙OH, leading to 81–93% degradation of various pollutants (RhB, phenol, atrazine, and ibuprofen) within 20 min over a wide pH range of 3–10, with >90% activity retained after five cycles (Fig. 7a).^21^ Chen's group reported that BiOCl piezocatalyst achieved an exceptional H_2_O_2_ production rate of 710 µmol g^−1^ h^−1^*via* dual pathways (water oxidation and oxygen reduction), and the integrated system delivered 70.7% antibacterial efficiency and 94.7% removal of sulfamethoxazole with a ten-fold kinetic enhancement compared to externally added H_2_O_2_, while also removing over 90% of various refractory pollutants across a wide pH range (2.0–10.0) when combined with periodate (Fig. 7b).^93^

**Fig. 7:**
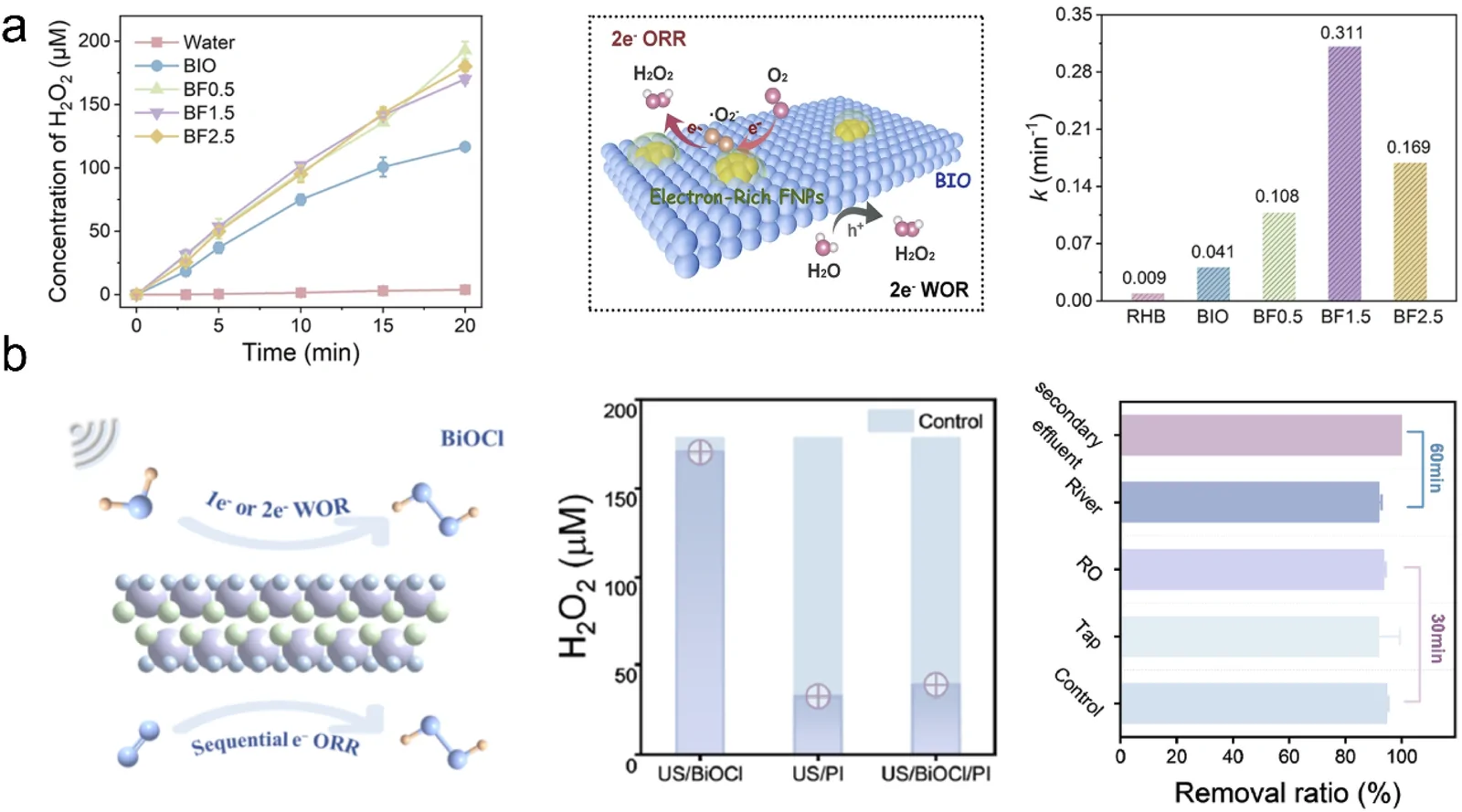
Time profiles of H_2_O_2_ evolution within 20 min in different systems, mechanism of H_2_O_2_ generation over BF1.5, RhB degradation with different catalysts and the corresponding pseudo-first-order kinetic constant within 15 min (a). Reproduced with permission from ref. 21 Copyright 2025 Springer Nature. Schematic overview of H_2_O_2_ generation pathways in BiOCl systems, quantitative determination of H_2_O_2_ concentration in systems US/BiOCl, US/BiOCl/PI, and US/PI and comparison with the control group and SMX degradation in different aqueous environments (b). Reproduced with permission from ref. 93 Copyright 2025 Elsevier.

### 4.3 Medical applications

Conventional therapies for malignant tumors and bacterial infections, such as chemotherapy, radiotherapy and antibiotics, often suffer from severe side effects and the emergence of drug resistance. Piezocatalytic therapy (PCT) harnesses mechanical energy (*e.g.*, ultrasound) to excite piezoelectric materials that generate ROS (including H_2_O_2_, ˙OH, and O_2_˙^−^) *in situ*, inducing oxidative stress that kills cancer cells or bacteria, while the weak piezoelectric electric field can also directly interfere with cellular electrophysiological activities. Compared with photodynamic therapy, ultrasound penetrates much deeper into tissues (>10 cm), and piezocatalysis remains effective even in the hypoxic, high-GSH tumor microenvironment. The *in situ*-produced H_2_O_2_ can act as a cytotoxic agent on its own, serve as a substrate for Fenton reactions, or function as an electron-sacrificial agent in radiodynamic therapy, greatly amplifying therapeutic efficacy. For example, tetragonal BaTiO_3_ (T-BTO) nanoparticles embedded in a thermosensitive hydrogel were intratumorally injected; under ultrasound (1.0 MHz, 1.0 W cm^−2^), the piezoelectric effect generated ˙OH and O_2_˙^−^, leading to complete tumor eradication in 4T1-bearing mice after three treatments, with no recurrence over 40 days and excellent biosafety (Fig. 8a).^94^ BiOCl@PAA nanosheets first produced H_2_O_2_ and ROS under ultrasound; subsequently, under X-ray irradiation, the *in situ*-generated H_2_O_2_ acted as an electron sacrificial agent to dramatically enhance the yield of ˙OH from radiodynamic catalysis. *In vitro*, the combination treatment caused the most severe DNA damage, and *in vivo*, three out of six mice bearing 4T1 tumors showed complete tumor regression, with intratumoral H_2_O_2_ levels first increasing and then decreasing, confirming the synergistic mechanism (Fig. 8b).^95^ A BTO/MoS_2_@CA core–shell piezocatalyst triggered ferroptosis by consuming glutathione and utilizing *in situ*-generated H_2_O_2_ to produce ˙OH, leading to lipid peroxidation and GPX4 inactivation, while simultaneously activating antitumor immunity (Fig. 8c).^96^ Au@BTO nanocomposites under ultrasound produced ˙OH and ^1^O_2_*via* a Schottky barrier, achieving >99% antibacterial efficacy against *S. aureus* and *E. coli* and promoting fibroblast and macrophage migration to accelerate infected wound healing (Fig. 8d).^97^

**Fig. 8:**
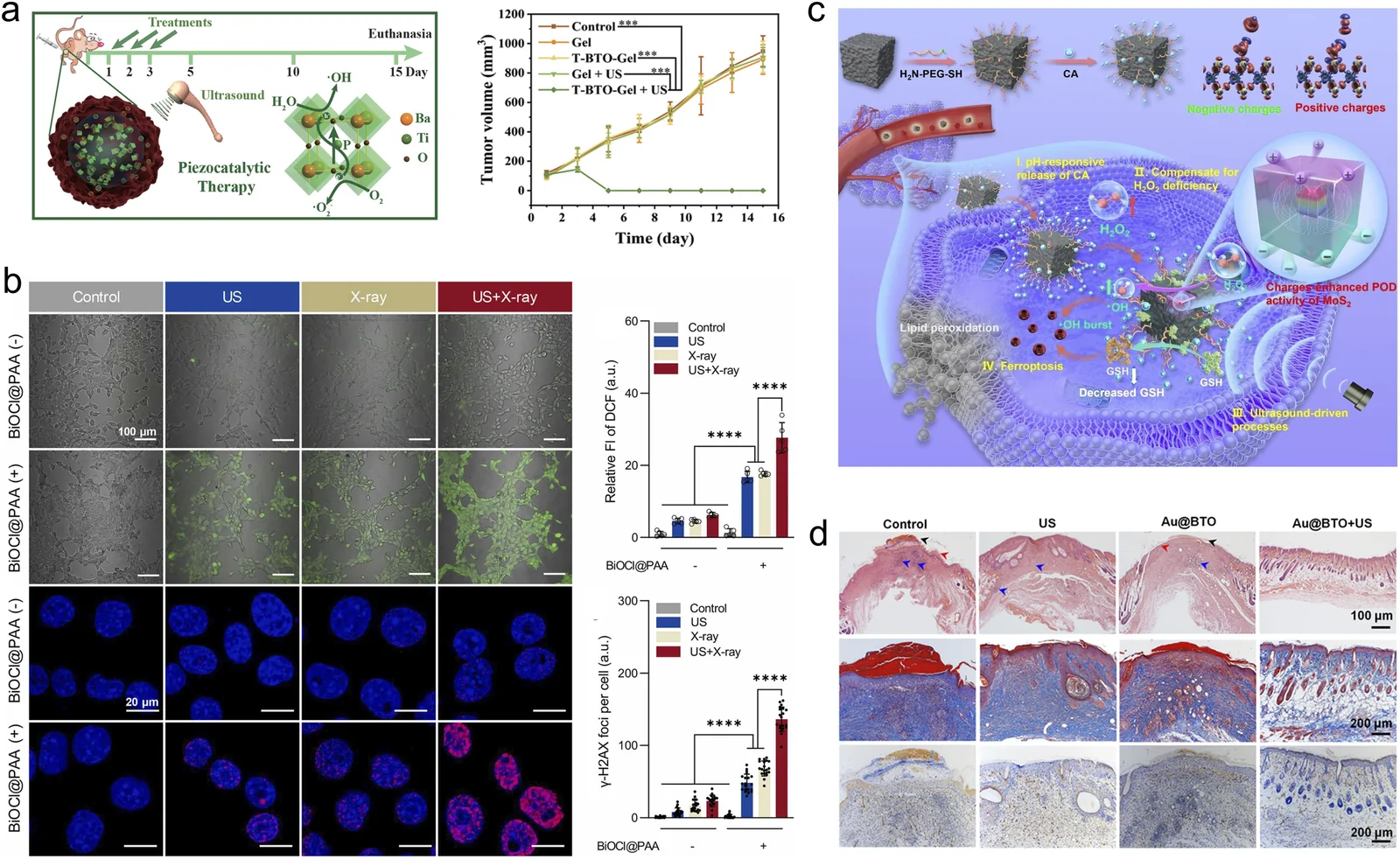
Schematic illustration of piezocatalytic therapy *in vivo* and time-dependent tumor-growth curves of tumor-bearing mice after different treatments, including the control group and gel group (a). Reproduced with permission from ref. 94 Copyright 2020 Wiley. DCF fluorescence images and relative fluorescence intensity of 4T1 cells upon different treatments, *n* = 5 (US irradiation: 1.0 W cm^−2^, 45 s) and fluorescence images and quantification of γ-H2AX foci in 4T1 cells, *n* = 20 (US irradiation: 1.0 W cm^−2^, 45 s) (b). Reproduced with permission from ref. 95 Copyright 2022 Elsevier. Schematic illustration of the synthesis of BTO/MoS_2_@CA and positive and negative charge promoted dissociation of H_2_O_2_ and piezoelectric catalytic therapeutic process of BTO/MoS_2_@CA under US irradiation (c). Reproduced with permission from ref. 96 Copyright 2023 Wiley. H&E staining, Masson's trichrome staining and immunohistochemical staining for CD31 expression of the tissue sections around wound regions collected on day 13 (d). Reproduced with permission from ref. 97 Copyright 2021 Elsevier.

## 5. Summary and outlook

### 5.1 Prospective integration with selective synthesis

H_2_O_2_ is widely used as a green oxidant in epoxidation, ammoximation, alcohol oxidation and C–H hydroxylation. However, its conventional use depends on concentrated H_2_O_2_ produced by the anthraquinone process, creating additional requirements for transportation, storage, dilution and stabilization. *In situ* generation followed by immediate consumption therefore offers a safer and more process-efficient route for fine-chemical synthesis. Piezocatalytically generated H_2_O_2_ has not yet been directly coupled with selective organic synthesis. The Au-Pd/TS-1 and PdFe/TiO_2_ systems generate H_2_O_2_ from H_2_/O_2_, whereas MC-3/TS-1 produces it electrochemically; these non-piezocatalytic systems are retained only as precedents demonstrating the value of local H_2_O_2_ production and immediate transfer to oxidation sites.^98–100^ Future piezocatalytic systems should couple mechanically driven H_2_O_2_ generation with selective consumption, suppress non-selective radical reactions and product degradation, and report H_2_O_2_ utilization efficiency and mechanical-energy cost alongside conversion and selectivity.

### 5.2 Priorities for piezocatalytic H_2_O_2_ production

Piezocatalytic H_2_O_2_ synthesis should be viewed not simply as a peroxide-production reaction, but as an integrated production–utilization platform in which H_2_O_2_ is generated, accumulated, activated and consumed at the point of use. Its principal value lies in replacing the transportation and dosing of concentrated oxidants with mechanically driven, on-demand chemistry. The field should therefore move beyond cataloguing new materials and recording production rates towards mechanistic certainty, comparable energy accounting and function-oriented reactor design.

Mechanistic discrimination remains the first priority. Ultrasound alone cannot establish a piezoelectric pathway because cavitation, local heating, contact electrification and sonochemistry may produce similar intermediates. Force-matched controls, non-piezoelectric analogues, isotope labelling, H_2_O_2_ mass balances and *operando* measurements are required to distinguish reactions driven by mobile carriers, surface-screening charges and secondary activation. Reporting should include catalyst loading, reactor volume, mechanical frequency, delivered power, temperature and atmosphere. Production rates should be normalized by both catalyst mass and liquid volume, whereas energy efficiency must be calculated using calibrated mechanical or acoustic energy rather than nominal instrument power.

Material design should focus on preserving accessible polarization and directing charges towards selective two-electron ORR and WOR pathways. Carrier screening should be treated as a controllable design parameter through regulation of carrier density, defects, interfaces and dipoles. Although high-power ultrasound is useful for mechanistic studies, it is unlikely to represent the practical endpoint. Ultrathin, flexible and porous piezocatalysts should instead be integrated into membranes, vortex reactors and continuous-flow systems capable of harvesting weak mechanical energy while avoiding catalyst recovery.

The decisive test is whether *in situ*-generated H_2_O_2_ provides a measurable advantage over an equivalent amount of externally supplied H_2_O_2_. Future studies should quantify its generation, accumulation, activation and consumption, while distinguishing direct piezocatalytic reactions from H_2_O_2_-mediated and secondary activation pathways. With standardized benchmarking, mechanistically grounded material design and application-specific reactors, piezocatalysis could develop from a laboratory proof-of-concept into a practical platform for decentralized water treatment, medical intervention and selective chemical synthesis.

## Author contributions

Zhi Li: conceptualization, investigation, writing – original draft, funding acquisition. Mingshan Zhu: conceptualization, supervision, writing – review & editing, funding acquisition.

## Conflicts of interest

There are no conflicts to declare.

## Data Availability

No primary research results, software or code have been included and no new data were generated or analysed as part of this perspective.
